# Perspective on “Active Brownian particles moving in a random Lorentz gas”

**DOI:** 10.1140/epje/s10189-026-00572-0

**Published:** 2026-04-02

**Authors:** C. Reichhardt, C. J. O. Reichhardt

**Affiliations:** Theoretical Division and Center for Nonlinear Studies, Los Alamos National Laboratory, Los Alamos, New Mexico 87545 USA

## Abstract

**Abstract:**

Self-propelled active matter can exhibit vastly different behavior than systems with purely Brownian motion. In Eur. Phys. J. E **40**, 23 (2017), Zeitz, Wolf, and Stark compared an active matter particle with a Brownian particle moving in a random obstacle array. They showed that near the obstacle percolation density, both Brownian and active particles exhibit the same subdiffusive behavior, but the active particle reaches a steady state more rapidly. They also found that for high activity, the active particle has a lower effective diffusion than the Brownian particle due to the increased self-trapping effect generated by the activity. This result opens new directions for the study of active matter in disordered media, including bacteria in porous media, active colloids on quenched disorder, and active particles in crowded environments.

**Graphical abstract:**

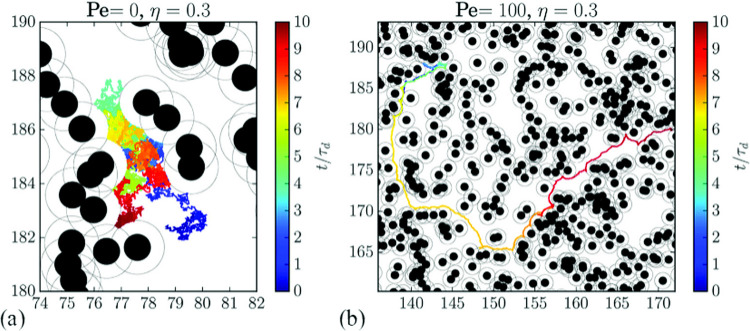

## Introduction

**Fig. 1:**
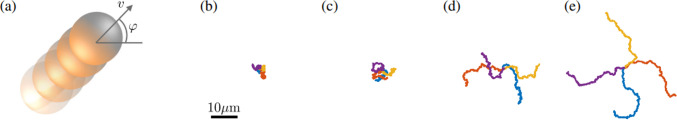
(**a**) Illustration of a two-dimensional (2D) active Brownian particle moving with speed *v* at an orientation of $\phi$. (**b**-**e**) Four representative 10 second particle trajectories for a particle with radius $R=1 \mu$m and viscosity $\eta =0.001$ Pa s in water at different velocities of (**b**) $v=0 \mu$m/s (Brownian limit), (**c**) $v=1 \mu$m/s, (**d**) $v=2 \mu$m/s, and (**e**) $v=3 \mu$m/s. From Fig. 2 of [3]

Active matter particles break detailed balance via some form of self-propulsion. The activity can take the form of run-and-tumble dynamics, or the particles can undergo driven diffusion in which the particle velocity remains constant but the direction of motion gradually changes over a characteristic persistence length [1–3]. The motion of a randomly moving particle can be characterized by its mean-square displacement over time, $\langle r^2(t)\rangle \propto D_\alpha t^\alpha$, where $D_\alpha$ is the generalized diffusion constant and $\alpha$ is the diffusive exponent. For a Brownian particle, $\alpha = 1.0$, but if the motion of the particle is slowed by trapping or crowding, then $0< \alpha < 1.0$ and the system is said to exhibit subdiffusion [4]. In the case of ballistic motion, $\alpha = 2.0$, and if collisions occur that cause the ballistically moving particle to change direction, superdiffusive behavior emerges for which $1.0 < \alpha \le 2.0$. An active particle generally undergoes superdiffusive motion on short or intermediate times, but at long times, its motion becomes diffusive due to the ever-changing direction of travel [1, 3]. Figure 1 shows an example of a driven diffusive or active Brownian particle moving in two dimensions (2D). The particle has a propulsion velocity of *v* and an orientation direction $\phi$ that gradually changes over time. Example trajectories for *v* values ranging from the Brownian limit of $v=0 \mu$m/s to $v=3 \mu$m/s appear in Figs. 1(b-e). As *v* increases, the trajectories are able to extend further from the $t=0$ position of the particle before the direction of motion becomes fully randomized. Both single active particles and assemblies of active particles have been extensively studied, but there are additionally numerous situations in which active particles can interact with some form of barrier or obstacle [3, 5–17]. One example of such a system is a bacterium in a disordered medium [18–21]. A natural question is how the motion of an active particle in a disordered environment, such as an assembly of obstacles, would differ from that of a Brownian particle. While the activity could lead to higher diffusion and larger displacements compared to a Brownian particle due to the correlated propulsion, the persistence in motion could also result in a self-trapping effect.

**Fig. 2:**
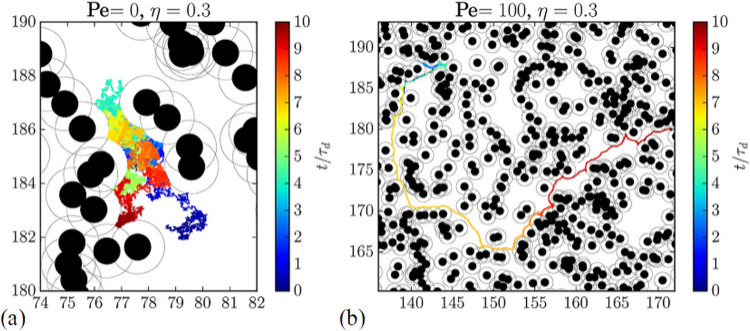
Particle trajectories with time $t/\tau _d$ encoded by color for (**a**) a Brownian particle and (**b**) an active Brownian particle in a system with a background obstacle density of $\eta =0.3$. From Fig. 1 of [22]

Zeitz *et al.* [22] considered an active particle moving through a random obstacle array, which is also known as a Lorentz gas [23]. A system of area *A* can be described in terms of the reduced density or the area covered by the disk-shaped obstacles, $\eta = \pi R^2/A$, where *R* is the disk radius. A percolation transition of the obstacles occurs near $\phi = 0.67$ [24] in 2D. Zeitz *et al.* focused on active and Brownian particles of finite size, such that the critical percolation occurs at a reduced density of $\eta =0.28$, and measured the mean-square particle displacements and effective diffusion constants as the obstacle density was swept upward through the percolation transition. For the Brownian particles, the motion is diffusive at lower $\eta$ values well below the percolation density, but close to the percolation density, the particles become increasingly trapped and the motion becomes sub-diffusive, similar to what is observed in a glass. Figure 2(a) shows the time-encoded trajectory of a Brownian particle moving through an obstacle array at $\eta =0.3$. For an active particle in the same array, Fig. 2(b) indicates that there are extended regions where the particle moves ballistically between obstacles.

**Fig. 3:**
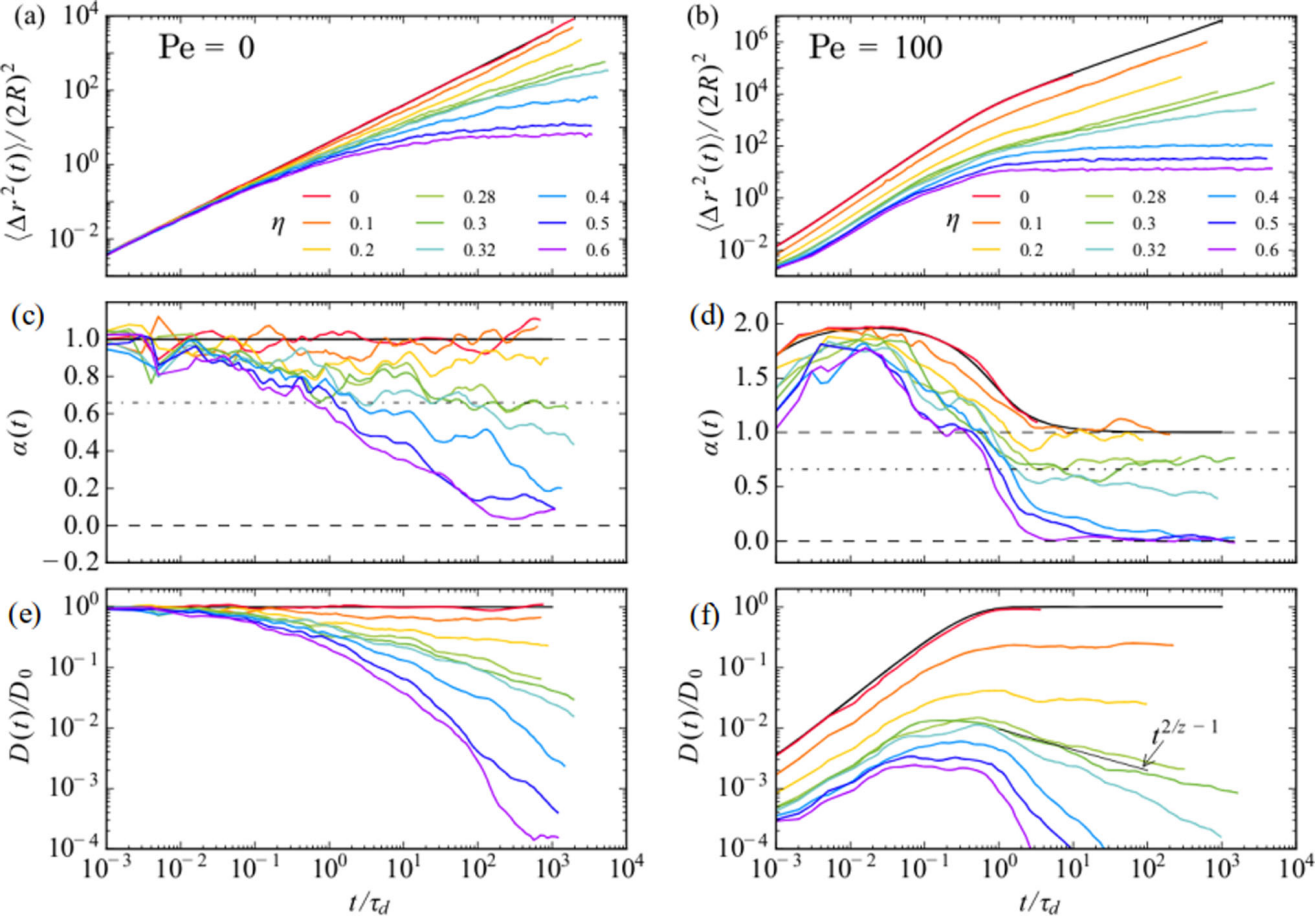
(**a, b**) Mean squared displacement $\langle \Delta r^2(t)\rangle /(2R)^2$, (**c, d**) local exponent $\alpha (t)$, and (**e, f**) local diffusion coefficient $D(t)/D_0$ versus scaled time $t/\tau _d$ for (**a, c, e**) a Brownian particle with $Pe=0.0$ and (**b, d, f**) an active Brownian particle with $Pe=100$ at obstacle densities ranging from $\eta =0.0$ to $\eta =0.6$. The percolation transition occurs near $\eta _c=0.28$. The active particles have an extended region at short times where the motion is superdiffusive, but for $\eta > \eta _c$ the motion of the active particles is reduced compared to the Brownian particles due to self trapping effects. From Fig. 2 of [22]

The motion of the Brownian particle is diffusive at low obstacle density but becomes subdiffusive as $\eta$ approaches the critical percolation threshold. A key advantage to the Lorentz gas model is that its percolation universality class is known, which gives a subdiffusive exponent of $\eta _c \approx 0.66$. Figure 3(a) shows the mean-square displacement versus time for a Brownian particle at varied obstacle densities from $\eta =0.0$ to $\eta =0.6$. The activity level is characterized by the Péclet number (*Pe*), with large *Pe* indicating high activity, so that Brownian particles have $Pe = 0.0$. The local diffusion exponent $\alpha (t) = d\log (\langle \Delta r^2(t)\rangle )/d\log (t)$ and the local effective diffusion constant $D(t)/D_0$ are shown in Fig. 3(c) and (e), respectively. For $\eta < \eta _c$, $\alpha (t) = 1.0$, indicating that regular diffusion is occurring. As $\eta$ approaches the critical $\eta _c$, $\alpha (t)$ approaches 0.66, indicated by the dashed lines in Fig. 3(c), while when $\eta >\eta _c$, $\alpha (t)$ approaches 0.0 and the diffusion constant monotonically decreases with time. Figure 2(b) shows the mean squared displacements versus time for the same system but with an active particle of $Pe = 100$. In Fig. 2(d), the corresponding local exponent obeys $\alpha (t) > 1.0$, indicative of superdiffusion, as expected for an active particle. In the $\eta =0$ system where there are no obstacles, the system shows superdiffusion at short times but has a crossover to regular diffusion at longer times. Near the critical density $\eta \approx \eta _c$, the active particles have an extended region of time in which $\alpha = 0.66$, indicating that the active particles are able to reach a steady state more rapidly than the Brownian particles. For $\eta > \eta _c$, the active particles eventually reach $\alpha (t) = 0.0$, and the decrease in *D* at long times is much more rapid than for the Brownian particles. These results indicate that when $\eta > \eta _c$, the active particles are actually less motile than the Brownian particles as the result of a self-trapping effect in which the active particle can become trapped behind an obstacle. The persistence of the active motion holds the active particle in the trapping site until the velocity has rotated far enough into a new direction to transport the active particle away from the trapping obstacle; however, the particle rapidly becomes trapped behind a different obstacle. Brownian particles constantly change their direction of motion and are therefore better able to explore the available free space compared to the active particles. Self-trapping of active particles has been observed in other active matter systems, including run-and-tumble systems, where for a given amount of substrate disorder there is often an optimal activity level that maximizes the motility of the active particles [6, 9, 12, 14, 16, 21].

**Fig. 4:**
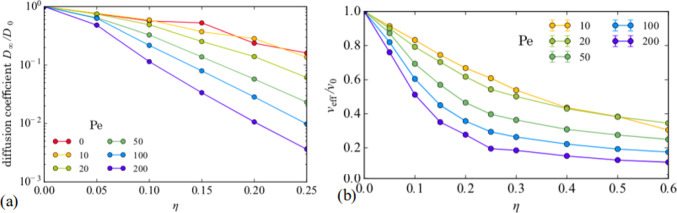
Diffusive and ballistic motions at different Péclet values. (**a**) The diffusion coefficient $D_\infty /D_0$ vs $\eta$, where $D_0$ is the $\eta =0$ diffusion coefficient. From Fig. 5 of Ref. [22]. (**b**) The effective propulsion velocity $v_\textrm{eff}/v_0$ vs $\eta$, where $v_0$ is the propulsion speed. From Fig. 7(a) of Ref. [22]

Figure 4(a) shows the long-time diffusion $D_{\infty }/D_0$ versus $\eta$ measured in the range $\eta <\eta _c$ for varied *Pe* [22]. Here, $D_0$ is the obstacle-free diffusion constant. For $Pe = 0.0$, the diffusion decreases by a factor of less than 10 as $\eta$ increases. In contrast, for $Pe = 200$, the diffusion drops by 300 times, illustrating the decrease in mobility caused by introducing activity. This behavior is underscored by the plots of the effective propulsion velocity $\eta /v_0$ in Fig. 4(b), where the velocity is higher at lower *Pe* where the particles are able to move through the free spaces of the obstacle assembly, whereas particles with large *Pe* spend a significant fraction of time immobilized in trapping sites behind obstacles.

**Fig. 5:**
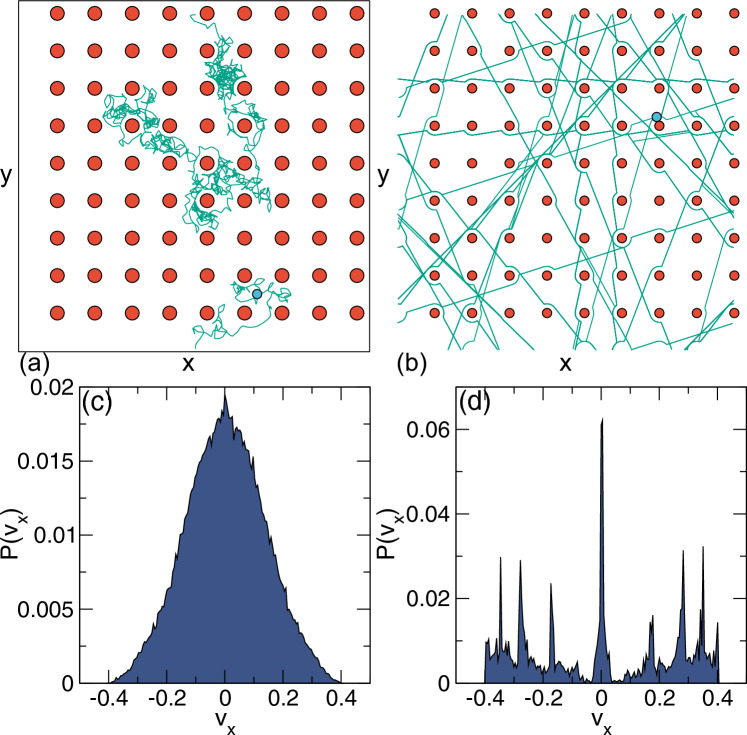
(**a, b**) Motion of individual particles through a periodic array of posts (red) with lattice constant $a_s$. (**a**) Trajectory of a Brownian particle. (**b**) Trajectory of a run-and-tumble active particle with run correlation length $l_a=20 a_s$. The motion is channeled along substrate symmetry directions. (**c, d**) The corresponding distribution of instantaneous *x* direction velocities $P(v_x)$ for (**c**) the Brownian particle from panel (**a**), where the distribution is Gaussian, and (**d**) the active particle from panel (**b**), where the peaks indicate the locking of the motion to the substrate symmetry directions. From Fig. 2 of [25]

A number of studies have built upon the work of Zeitz *et al.* For example, the active particles can move through periodic arrays of obstacles [26–29]. A Brownian particle traversing a periodic obstacle array, as illustrated in Fig. 5(a), diffuses between the obstacles and has the Gaussian velocity distribution $P(v_x)$ shown in Fig. 5(c). The case of a run-and-tumble active particle is shown in Fig. 5(b). Here the particle travels ballistically for long distances between the obstacles along preferred symmetry directions of $\theta _m=0^\circ$, ${45}^\circ$, and $90^\circ$. As the radii of the obstacles decreases, the number of available easy flow directions increases, and for the square lattice shown in the figure, these correspond to motion along $\theta _m= \tan ^{-1}(n/m)$, where *n* and *m* are integers. The active particle motion remains superdiffusive out to longer times compared to a system with randomly placed obstacles, and the velocity distribution $P(v_x)$ is highly non-Gaussian and contains peaks associated with motion along the easy flow directions of the substrate, as shown in Fig. 5(d) [27].

**Fig. 6:**
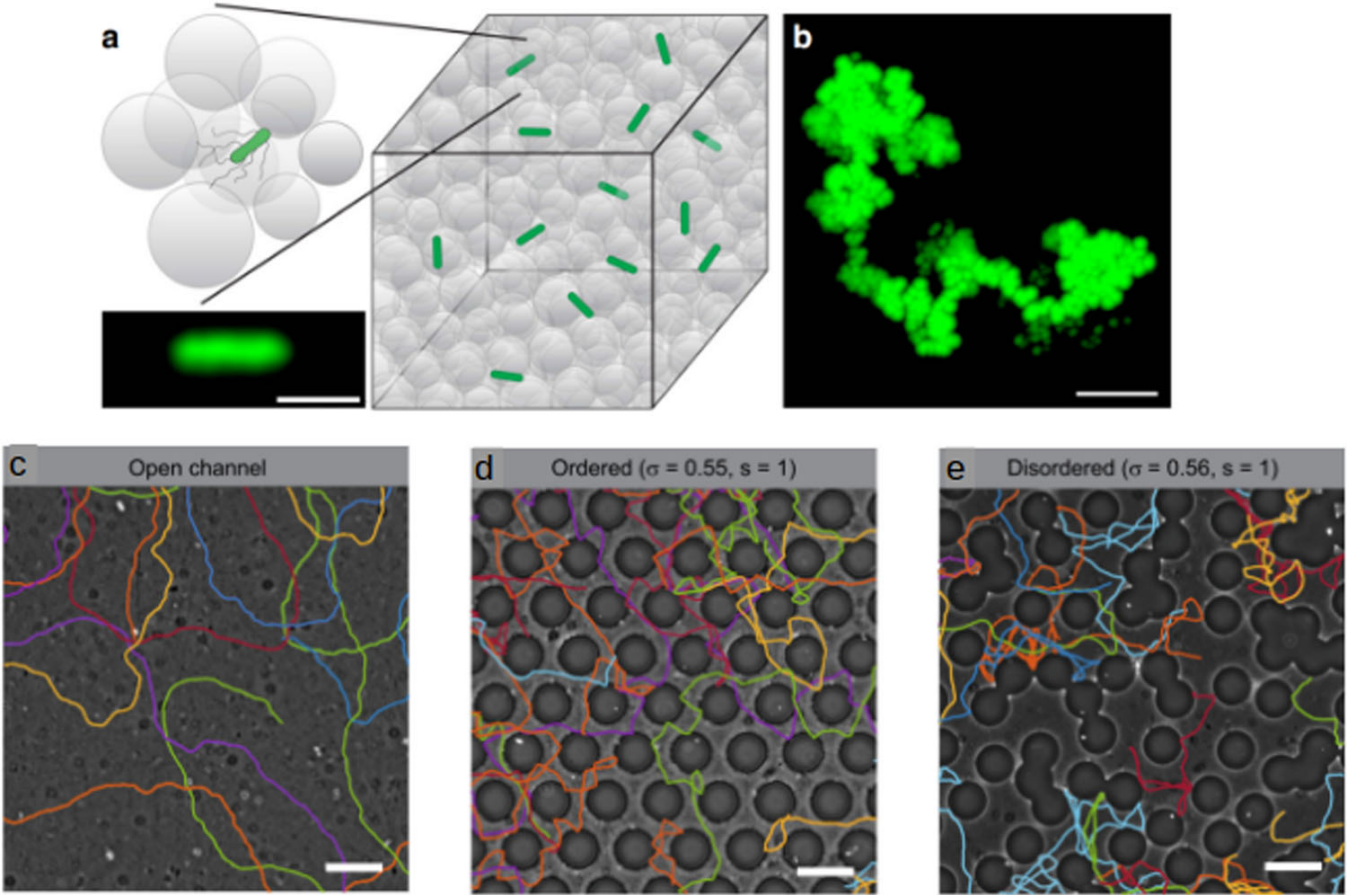
(**a,b**) Bacteria moving in a three-dimensional (3D) array of hydrogel spheres, shown (**a**) schematically and (**b**) from fluorescent imaging of a tracer particle. From Fig. 1 of [18]. (**c,d,e**) Bacterial motion in (**c**) smooth, (**d**) triangular, and (**e**) disordered obstacle arrays. From Fig. 1(**a,b,c**) of [29]

There have now been numerous experimental realizations of the Zeitz *et al.* model. Figure 6(a,b) shows a three-dimensional (3D) hydrogel containing tortuous paths for bacteria motion. Here, the bacteria exhibit intermittent hopping from pore to pore of the medium, and there is a crossover from superdiffusion at short times to regular diffusion at long times, with the net diffusion decreasing with increasing obstacle density [18]. Figure 6(c,d,e) shows experimental trajectories for bacteria moving in 2D on a smooth substrate, a triangular obstacle array, and a random obstacle array, demonstrating different modes of motion. In this case, for shorter times, the motion on the ordered arrays shows locking along symmetry directions of the substrate; however, at longer times, the motion for both ordered and random arrays exhibits similar behavior [29].

## Future directions

As active matter becomes an increasingly mature field, there will be more interest in studying active matter systems with increased complexity in terms of the rules of how the particles move, interact with each other, or transport themselves through random or structured environments. Here, we highlight some possible future extensions for the Zeitz model: (1) The active particle could change its behavior over time. For example, if the active particle reaches a state where it is no longer moving due to trapping, it could decrease its Péclet number for a fixed time in order to escape the trapping site. Conversely, if the particle is moving rapidly for extended periods, it could increase its Péclet number to amplify the motion, or it could reverse or otherwise modify its swimming direction. In this way, the particle could optimize its motion via feedback. (2) The spherical active particles could be replaced by more complex particle geometries, such as polymers, rods, flexible chains, or deformable active particles moving through obstacles. There has already been some work treating active polymers in disordered media [30]. (3) Variations of the model could be applied to the spreading of infections. Some work has already been done on epidemic transmission through active matter systems [31], but it would be interesting to explore the effective diffusion in an infectious model. (4) The particles and/or the environment could be made chiral. Some groups have considered chiral active particles in obstacle arrays [32–34]. These systems could have interesting topological effects or edge modes. (5) Different substrate geometries could be introduced, such as disordered hyperuniform, fractal, quasiperiodic, or anisotropic. (6) Models could be considered in which the obstacles themselves are dynamical and can move, either independently or in response to the motion of the active particles. (7) Collective effects could be captured by considering multiple interacting active particles. (8) The active particles could move over different types of complex networks [35].

Many of the studies performed to date have focused on diffusion and diffusion exponents, but there are also many other quantities that could be explored, such as aging effects and memory. Often studies are performed on systems where there is no external drive on the active particles; however, it would be possible to add a drift force, which could arise from a fluid or an applied field. In the case of active particles in a fluid, additional hydrodynamic effects can arise, so in the presence of a porous medium, the medium itself could induce hydrodynamic flows with which the active particles could interact. In this case, there could be hydrodynamic-induced trapping effects, or alternatively the hydrodynamics might cause the particles to avoid entering trapping sites. In addition to applying dc flows or fields, the particles could be subjected to ac driving, or to a combination of ac and dc driving, which could increase the diffusion.

Many active matter studies related to the Zeitz model have been performed in 2D, but a number of real-world applications would be in 3D, which would give a different percolation threshold. Also, studies in both 2D and 3D have focused on impenetrable or penetrable obstacles; however, the substrate could have other topological features, as in the case of gels or clusters, where there could be multiple percolation transitions for different densities or where the percolation could occur anisotropically and appear first in one direction and then in another. Instead of non-penetrable obstacles, the system could contain pinning sites, localized changes in the effective friction, or random landscapes where active particles would be able to hop over higher energy barriers. In addition to varying the density of the obstacles, it would also be possible to change the nature of the interaction of the particles with the obstacles. A particle could move along a wall, reflect from it, be attracted by it, or be repelled by it; it is also possible to modify the roughness of the walls or introduce increased or decreased friction for a particle that is sliding or moving along a wall. In some cases, the interaction with the walls could change the propulsion mechanics, causing active particles to move faster when in contact with a wall. If the obstacles are able to move, additional percolation transitions could occur, with one transition associated with contact percolation at a low density, and another associated with a jamming transition at higher density.

## Summary

Active matter is a rapidly growing interdisciplinary field with applications to biological systems, statistical physics, soft matter, social systems, and robotics. Early studies focused on single or interacting particles moving over a smooth substrate. Zeitz *et al.* [22] introduced a model for an active particle moving on an obstacle array to create an active matter version of the Lorentz gas. Despite the apparent simplicity of this model, Zeitz *et al.* found that it could exhibit a number of interesting effects, such as showing the same behavior near percolation as Brownian particles. Additionally, the activity can actually decrease the diffusion in these systems by causing increased trapping. The model of Zeitz *et al.* can be applied to a wider range of active matter systems, including run-and-tumble particles, active particles on periodic substrates, and even deformable substrates, and there have been a number of theoretical extensions and experimental realizations of this system. These include active colloids on patterned substrates, swimming bacteria in 2D and 3D systems, ordered and disordered substrates, active polymers, and active motion on periodic substrates. A growing number of new research directions have been inspired by the work of Zeitz *et al.*, which will lead to more studies of active matter in complex environments, the development of controlled modes of motion for active particles, and the creation of increasingly complex environments. The work of Zeitz *et al.* also nicely brings together established ideas from statistical mechanics, such as percolation, with new ideas in nonequilibrium systems and active matter.

## Data Availability

No datasets were generated during and/or analyzed during the current study.
